# Mobility, Nonstationary Density, and Robbery Distribution in the Tourist Metropolis

**DOI:** 10.1007/s10610-022-09528-4

**Published:** 2022-09-01

**Authors:** Riccardo Valente, Juanjo Medina-Ariza

**Affiliations:** 1 Department of Geography, University of Rovira I Virgili, Tarragona, Spain; 2 Department of Criminal Law and Crime Sciences, University of Seville, Seville, Spain

**Keywords:** Robbery, Mobility, Density, Ambient population, Tourism pressure

## Abstract

This study looks at the spatial distribution of robbery against residents as a function of nonstationary density and mobility patterns in the most densely populated city in Spain, Barcelona. Based on the geographical coordinates of mobile devices, we computed two measures of density of the ambient population and the tourist presence, for work days, weekends, and holidays in 2019. Negative binomial regressions are then estimated to analyse whether these measures are correlated with the risk of robbery, controlling for land use and the characteristics of the social environment. The model reveals that residents’ chances of being exposed to robbery in Barcelona depend on the social relevance and tourism attractiveness of certain places at particular times of the year. Our results disclose two sources of social disorganization as stronger predictors of the occurrence of robbery in Barcelona, respectively linked to structural processes of residential instability and daily and seasonal mobility patterns. On the one hand, we found that the effect of the density of international tourists on the outcome variable is mediated by residential volatility, which is assumed to be associated with housing shortages in neighbourhoods where short-term vacation rentals are widespread. On the other hand, the ability to exert effective social control is significantly undermined in urban areas, where the ambient population and the volume of tourists outnumber the resident population, thus increasing incidents of robbery victimization. The implications of these findings for urban policy and crime prevention in the Catalan capital are discussed.

## Introduction

On a daily basis, urban dwellers move out their places of residence and are replaced by people from other parts of the city, at the same time that commuters living outside the city and international visitors increase the size of the population still further. Among other things, these floating populations have an impact on the spatial and temporal patterning of crime (De Nadai et al., 2020; Haleem et al., 2021; Lee et al., 2021; Oliveira et al., 2018). The objective of this study is to analyse how population density varied throughout 1 year (2019) as a function of the combined pressure of the mobility of residents and visitors and how these changes might be linked to the risk of being robbed in the city of Barcelona, Spain. We computed two measures of density of the ambient population and the tourist presence using mobile phone location data and used them to test the hypothesis that the non-stationary densities of the local ambient population (i.e., the sum of the resident population with the population entering this area, minus the population leaving the same area) and of international visitors are associated with a greater chance of residents becoming victims of robbery. According to our results, the presence of tourists is saturating the city’s carrying capacity by sharpening a structural problem of overcrowding. In such situations, robbery emerges as a negative externality, especially in areas with greater residential instability.

The paper is structured as follows. The first section summarizes current advances in the field of crime and mobility studies with a focus on the concept of the ambient population, which synthetizes scholars’ efforts to overcome the limitations of using only the resident population as the denominator of the crime rate. The current situation with the relationship between land use, population density, and crime is addressed in the second section. The case study is presented in section three, followed by a description of data and methods used, and the results. The last section is dedicated to the conclusions and a discussion of the possible implications of our findings, which, although limited to the pre-pandemic period, might inform debates on the present-day recovery.

## Urban Mobilities and the Ambient Population at Risk of Victimization

A review of the most recent literature in criminology discloses growing consensus on the need to integrate measures of human mobility into crime studies (Browning et al., 2021). However, as the operationalization of the concept of mobility is costly, most of the earlier analyses have been limited to the use of a static measure of the residential population to estimate the impact of crime in our cities. This approach fails to provide accurate estimates of the population that is actually at risk of becoming victims of crime, especially for some types of crime such as assaults (Boivin, 2013), robbery (Zhang et al., 2012) and other forms of violent crime (Andresen, 2011). Problems arise because urban spaces are increasingly crossed by floating populations (commuters, visitors, and residents themselves during their daily activities), which reduces the value of static measures of spatial occupancy. This is particularly relevant in relation to the central commercial, entertainment, and working districts of large metropolitan areas or in areas with a large transport node. This issue is not entirely new, and it has been discussed ever since the 1960s (Boggs, 1965; Harries, 1991), although computations using alternative measures of the population at risk have been long unfeasible due to the lack of suitable data. Today, however, the increased availability of new forms of data and the development of geographical information systems have eased this task, enabling a branch of the literature to converge on the notion of an “ambient population,” that is, the population that is present in a particular spatial unit at a given moment (Andresen, 2011; Haleem et al., 2021; Lee et al., 2021; Malleson & Andresen, 2016). Measures of the ambient population have been extracted from a very heterogenous set of data sources, including social media, cell towers, transportation surveys, or mobile-phone tracking (Boivin & Felson, 2018; De Nadai et al., 2020; Felson & Boivin, 2015; He et al., 2020; Hipp et al., 2019; Johnson et al., 2021; Lan et al., 2019).

The rationale behind these efforts is rooted in the idea that both crime and mobility are spatially and temporarily patterned phenomena, such that, if we can track individuals’ movements in their daily routines, we are in a good position to predict when and where crime will happen. Therefore, the emphasis on the ambient population is framed in terms of two mainstream criminological theories. On one hand, according to routine activity theory (Cohen & Felson, 1979), crime occurs when at least three conditions converge simultaneously in space and time: the co-presence of attractive targets and motivated offenders, along with the lack of capable guardians. Mobility patterns then become key to anticipating these conditions, as crime is shown to be more likely to happen in close proximity to those facilities where people tend to concentrate their activities (workplaces, leisure facilities, commercial premises, transit nodes, etc.) (Bowers, 2013; Felson & Eckert, 2018; Kinney et al., 2008; Summers & Johnson, 2017; Wang et al., 2017). Along the same lines, Brantingham and Brantingham (1995) drew a distinction between places that might function as either “crime attractors” or “crime generators,” thus increasing criminal opportunities. On the other, taking social disorganization theories into account (Bursik & Grasmick, 1993; Sampson, 2012; Sampson & Groves, 1989; Shaw & McKay, 1942; Yuan & McNeeley, 2017), the social and ecological characteristics of places, including population density, residential instability, and the unequal distribution of income and opportunities, all affect community integration and informal social control. In this regard, land use and the availability of public spaces in which to socialize (“palaces for the people”: see Klinenberg, 2018) become key to fostering a shared belief that the members of a community will intervene if norms are broken and will provide social control.

## Mixed Land-Use, Population Densities, and Outdoor Crime

The way public space is designed and used significantly influences the spatial and temporal patterning of crime. In a pioneering study of US cities published by Jacobs (1961), mixed-use neighbourhoods that combine residential and commercial facilities enhance the use of outdoor spaces, thus increasing social control over deviant behaviour. Conversely, Stark (1987) supports the idea that mixed-use neighbourhoods create more opportunities for crime because of their walkability and the greater likelihood of the co-presence of potential victims and offenders in a context of increasingly absent guardianship. In a similar vein, Taylor (1988) argued that extensive commerce reduces social control, thus creating the conditions for crime to occur.

Indeed, the nature of the relationship between the socio-ecological features of urban spaces and crime depends on several factors, including the specifics of the place under scrutiny and the type of crime being addressed. Looking at recent contributions estimating the risk of outdoor crime — including robbery, on which the present analysis focuses — commercial land-use was found to be associated with a higher chance of becoming the victim of violent crimes (e.g. assault, robbery or homicide) (Boessen & Hipp, 2015). A study of violent crime in Los Angeles concluded that a mixture of land-uses reduces the risk of becoming a victim of crime (Anderson et al., 2012), while a more recent analysis by Inlow (2019) found that mixed land-uses had a positive association with homicide in Portland, Oregon. Using data from Coquitlam (Canada), Wuschke and Kinney (2018) conclude that commercial areas, particularly shopping centres, are associated with a disproportionate amount of crime. Overall, as anticipated in the previous section, it is widely agreed that urban areas that bring crime attractors and crime generators together increase the chances of one becoming a victim of crime (Twinam, 2017; Williams & Hipp, 2019; Wo, 2019; Zahnow, 2018), while according to Bernasco and Block (2011), one can also expect spillover effects on neighbouring areas.

Conversely, the exact nature of the relationship between population densities and crime has proved more controversial. According to Battin and Crowl (2017, p. 138), “density and crime have been shown to have no correlation or a strong and significant correlation depending on what and how it was measured.” Analyses within the framework of social disorganization theory have shown that robbery is significantly correlated with structural density, defined as the proportion of multiple dwellings in an area (Sampson, 1983), while more generally several scholars have indicated that dense, mixed-use neighbourhoods tend to increase pedestrian traffic and to reduce the likelihood that street users will know one another (Brueckner & Largey, 2008; Nolan, 2004; Zhang & Peterson, 2007). Overcrowding has also been shown to reduce residents’ willingness to manage nearby outdoor spaces (Kurtz et al., 1998; Wikstrom, 1991), which ultimately leaves room for unlawful behaviour. However, the positive association between density and crime has been questioned by other authors, who failed to find any correlation (Li & Rainwater, 2000) and concluded that population density has no or very little influence on violent crime, and only a negative relationship with property crime (Battin & Crowl, 2017).

## The Case Study: Barcelona, a Densely Populated City with an Excess of Tourists

According to Rae’s (2018) estimates, Barcelona has the most densely populated urban areas in Spain and the most densely populated major country in Europe. With an average of 15,992 inhabitants per square kilometre, the population distribution varies strongly across neighbourhoods, with the densest areas located in the inner-city district of *Ciutat Vella* tripling this figure. As Aibar and Bijker (1995) explain, the historical legacy of the Catalan capital has much to do with the present-day urban layout of the city centre. In fact, *Ciutat Vella* (literally, the Old City) was surrounded by a wall built after a defeat at the hands of Bourbon troops in 1714 and was not demolished until the second half of the twentieth century. As a result of this geographical restriction, population density in Barcelona, with 856 inhabitants per hectare, was even then the highest in Spain and one of the highest in Europe (the population density of Paris, for instance, was under 400 inhabitants per hectare). In the 1860s, the Catalan engineer Ildefons Cerdà designed an “extension” (*Eixample* in Catalan) to the city by installing the iconic grid pattern crossed by the wide avenues and square blocks that characterize present-day Barcelona. Cerdà’s plan was to combine the extension outside the wall with a renovation of the old city. However, “the city council, trying to avoid any conflicts with the powerful property owners of the old city, preferred to support projects that kept the reforms in the old city to a minimum” (Aibar & Bijker, 1995, p. 10). During the succeeding 50 years, the city grew gradually by annexing neighbouring municipalities, which gave it its present-day shape.

In recent decades, Barcelona has undergone a further extensive urbanization project, especially after being selected to host the Olympic Games in 1992. This occasion was the driving force for a new stage in the modernization of the city’s urban landscape and infrastructure. This included the declining areas of the city centre, which attracted private capital investment and inaugurated a project of urban renewal called the “Barcelona model,” considered a perfect example of public–private cooperation. Tourist activity has been growing exponentially ever since, reaching a record number of 11.9 million visitors in 2019, that is, an average of 32,000 people travelling to the city every day. In a city of 101.3 km^2^, where tourist attractions and accommodation are concentrated in a smaller part of its surface area, this creates huge pressures on resident’s daily routines. For example, Cocola-Gant and López-Gay (2020) linked tourism-led gentrification with residential displacement in those areas where there is a lack of balance between tourism facilities and other land-uses. Garcia-López et al. (2020) found that the growth in rental prices was correlated with the spread of Airbnb across the city. Arias-Sans and Russo (2016) focused on the creation of tourism-only areas in the city, areas that are no longer publicly accessible, such as Park Güell. The effect of tourism on crime has also been addressed by Maldonado-Guzmán (2020), who highlights the significant relationship between Airbnb lodgings and property crime in Barcelona.

Despite the fact that studies of crime and tourism in Barcelona are sporadic, the international literature suggests that the growth in tourism is increasing the opportunities for crime, especially economic crimes (Biagi & Detotto, 2014; Harper et al., 2012; Mataković, 2020; Mawby, 2010; Xu et al., 2018). Tourists are often seen as carrying valuable items (including cash) and therefore as lucrative targets. Moreover, they generally lack a comprehensive knowledge of the environment, reducing their ability to handle unforeseen situations. Finally, visitors can be seen as outsiders within the city they travel to, people who are “outside of their normal routines, altering the convergence of victims and offenders regardless of who they might be (i.e., local resident or visitor)” (Drawve et al., 2020, p. 440). Using Spanish data, Montolio and Planells-Struse (2013) estimated that a 1% increase in the number of tourists increases serious property crimes by 0.35% and minor property crimes by 0.10%.

## Data and Methods

### Territorial Scope

The analysis below focuses on the city of Barcelona (Spain), which had a population of 1,636,762 on 1 January 2019. Although focusing on a single city has its limitations, Barcelona’s geographical features mark out a relatively autonomous and bounded area due to the presence of natural barriers (e.g. the natural parks of Collserola to the north and Serralada de Marina to the north-east, and the Mediterranean Sea to the south) and artificial features (e.g. the Zona Franca and the airport to the west, the B-20 motorway running from north to east, and the intersection of the B-20 with the C-33 and C-58 to the north-east). For the purposes of this study, the city’s territory is divided into a combination of administrative neighbourhoods and mobility cells, giving a total of 63 geographical units. According to the technical specifications of the Spanish National Institute of Statistics (INE, 2020), a mobility cell is a geographical unit of at least five thousand residents, a threshold ensuring that mobile-phone operators can provide sufficient information to explore mobility patterns (inflow and outflow) across cells. As for the case of Barcelona, while mobility cells often overlap with neighbourhoods, there are exceptions that required merging a few of them to match the city’s administrative divisions. These pre-processing steps enabled the creation of a dataset capable of integrating neighbourhood-based information about the main features of the built environment and the characteristics of particular areas.

### Outcome Variable: Robberies in 2019

In 2019, a total of 12,914 robberies were reported to or recorded by the Catalan regional police, the *Mossos d'Esquadra*. According to the Spanish Penal Code, robbery is classified as a crime against property implying the use of violence or threats. Crime data give the geographical coordinates of each incident, allowing robberies to be mapped using geographical information systems and the sum in the corresponding geographical unit of analysis to be computed. Given that mobility correlates were available only for specific dates, as detailed in the subsequent section, crime data were aggregated at three different times of the year: public holidays, including the peak summer season (22 July to 23 August) and Christmas holidays (21–31 December), weekdays (excluding official public holidays) during working hours (10:00–18:00), and weekends (Saturdays and Sundays).

### Measures of the Ambient Population

In an attempt to arrive at an accurate measure of the population exposed to the risk of becoming victims of robbery in Barcelona, this study explores the value of a new data set provided by the Spanish National Institute of Statistics (INE, 2020) within the framework of a pilot study of mobility based on mobile-phone locations. Using the spatial and temporal locations of mobile phones of the three biggest mobile-phone operators in Spain (Movistar, Orange and Vodafone, with together 78.7% of market share), INE created a set of origin and destination matrices enabling the analysis of mobility patterns (inflow and outflow) across mobility cells. These matrices are available for the working hours (10:00–18:00) of an ordinary week in 2019 (18–21 November), the weekend of the same week in November, and three more days selected as proxy measures of seasonal peaks in summer (20 July and 15 August) and at Christmas (25 December).

The origin and destination matrix incorporates the displacements from an area of residence to a destination area. The area of residence is defined as the geographical unit where a mobile phone is located most of the time between 00:01 and 06:00, based on a 60-day tracking record. As for the procedure for determining the area of destination, methods differ slightly depending on the matrices. Taking a week in November as a reference, the area of destination is the geographical unit where the mobile phone is located between 10:00 and 18:00, provided that it stays in the same area for at least four hours a day and two days out of the four observed. At the time of estimating the volume of seasonal population (July, August, and December), the area of destination is the geographical unit where individuals stay overnight (i.e., the most recurrent location between 22:00 on the day of scrutiny and 06:00 the next day).

As a result, mobility data for the working week in November provide accurate information to identify the locations of people throughout the 10:00–18:00 time period and, accordingly, to compute the day-time ambient population, the latter being the result of the sum of the residential population with the population entering this area, minus the population leaving the same area (Haleem et al., 2021). The seasonal estimates are less accurate, but they are still suitable for estimating the volume of people who stay overnight and who are most likely to occupy the public space at some point in the day. The ambient population was divided by the size of the area to estimate the effects of exposed population densities on the outcome variable.

One of the drawbacks of the INE’s dataset is that it only captures displacements of residents in Spain: that is, no data are available for mobile phones used while roaming, thus preventing any analysis of the mobility of tourists. To fill this gap, we were able to access an alternative source of mobile phone data from Vodafone’s users available at the geographical level of 500 × 500 m grids, with a breakdown by days and 8-h blocks during 11 months between April 2019 and February 2020. Relying on its market share (approximately 23%), Vodafone estimates the total number of people, including those from other mobile network operators, who passed by each grid. The dataset allows for a discretization between “sporadic” and “frequent” users of public spaces in Barcelona, which is elaborated by the data owner itself based on the observation of people’s displacements over a period of 30 days to calculate the probability of a person being localized in a given area. We used the category of “sporadic international users” to elaborate our measure of the tourism density. To this end, we first calculated the percentage of sporadic international users who crossed the grids out of the total users for each corresponding time period, and then used this value to account for the proportional share of the INE’s local ambient population that can be classified as international visitors. Finally, we imputed grid-level data to the 63 units in our analysis.

Finally, a measure of demographic pressure on the resident population is also included in the model. This is equivalent to the quotient of the division between the total volume of the ambient population (including sporadic international visitors) and the resident population. The assumption here is that higher values of demographic pressure might be associated with lower social control, as non-residents outnumber residents.

### Characteristics of Built and Social Environments

It was hypothesized that land-use measures may have an impact on the likelihood of becoming the victim of a robbery in Barcelona. To test this assumption, a land-use mixture index was calculated based on Frank et al. (2006), and a ratio of commercial-to-residential land-use to establish a difference between land-uses. In order to control the results for the socio-ecological features of the geographical units under analysis, four variables were used. The first was a composite index of residential instability, calculated as the average of two standardized measures of the proportion of residents living in the same area for more than 5 years (reverse coded), and the rate of internal changes of residence per 1000 inhabitants (Hipp & Roussell, 2013; Inlow, 2019). The second was the income quintile share ratio (S80/S20) as a measure of economic inequality, calculated as the ratio of total income owned by the population in the top income quintile to that received by those in the bottom quintile. The third was the mean age of the resident population in each unit of analysis, and the fourth the size of the resident population.

### Analytical Approach and Statistical Processing

The objective of the proposed analysis is to assess the influence of population density, the characteristics of the built environment, and its intended use upon the chances of robbery against residents occurring in the city of Barcelona. With this objective in mind, and considering significant overdispersion when using Poisson models, a negative binomial regression model (hereinafter referred to as NBM) was chosen. The baseline model (model 1) first tests the influence of the two measures of exposed population density on the likelihood of becoming the victim of a robbery, one derived from the sum of the resident population with the population entering this area minus the population leaving the same area, the other calculated using a proxy measure of the presence of tourists. Model 2 introduces the built environment and land-use. The last model (Model 3) includes control variables in order to account for the possible influence of the features of the social environment. The same structure applies to weekdays (a), weekends (b), the summer and Christmas holidays (c). All correlates were standardized (*z*-scores) before running the model. Descriptive statistics are provided in Table 1.

**Table 1:** Descriptive statistics

	Min	Max	Mean	SD
Robbery, weekdays (10:00–18:00)	0	152	31.3	27.8
Robbery, weekends	3	413	70.4	84.5
Robbery, public holidays	0	99	19.6	20.9
Land-use mix	.29	.96	.66	.18
Commercial-to-residential ratio	.04	.22	.10	.03
Ambient population (density), weekdays	2541	50,195	26,583	13,435
Ambient population (density), weekends	2077	55,635	25,838	13,934
Ambient population (density), public holidays	1589	44,508	22,853	12,008
Sporadic international visitors (density), weekdays	121	9114	1957	1940
Sporadic international visitors (density), weekends	158	10,538	2625	2503
Sporadic international visitors (density), public holidays	142	10,035	2521	2339
Residential instability index	− 1.36	3.92	.00	.89
Income quintile share ratio	2.10	3.68	2.83	.31
Age (*x̄*)	37.8	48.64	44.04	2.07
Resident population size	6884	58,642	25,990	13,315

### Robustness Checks

Four sets of robustness checks have been implemented to test the hypothesis of a significant relationship between non-stationary densities and crime by running alternative specifications of the negative binomial model. In a first set, we used theft against residents (e.g. “pickpocketing”) instead of robbery as the dependent variable. We narrowed the focus on non-resident victims in a second set of tests, using both theft and robbery. A third check involved the substitution of our original measures of population density with an alternative measure extracted from OpenCelliD (Johnson et al., 2021). The results of the robustness testing are discussed in the following section and fully disclosed in Annex I. Finally, we also run models using logarithmic transformations of the counts of robbery tested for spatial dependence in the residuals to explore whether we need to account for autocorrelation. Tests for spatial dependence are available upon request.

## Results

### Descriptive Analysis of Robbery Data

A descriptive analysis of the temporal distribution of robbery in 2019 reveals that 17% occurred on workdays between 10:00 and 18:00. The relative majority of robberies (38.6%) over a week were concentrated at weekends. Breaking down the data by months, the distribution of robberies is not significantly different across the year. In 2019, robberies against tourists represented 4.3% of the total. In line with the previous literature (Weisburd et al., 2016), robbery is strongly concentrated in a small area of the city, as more than half of all robberies take place in ten out of the 63 geographical units under analysis. Using a smaller unit of analysis, the spatial concentration of robberies increases: one tenth of the census tracts accounts for 55% of all robberies in 2019.

### Mobility Patterns in Barcelona

**Fig. 1:**
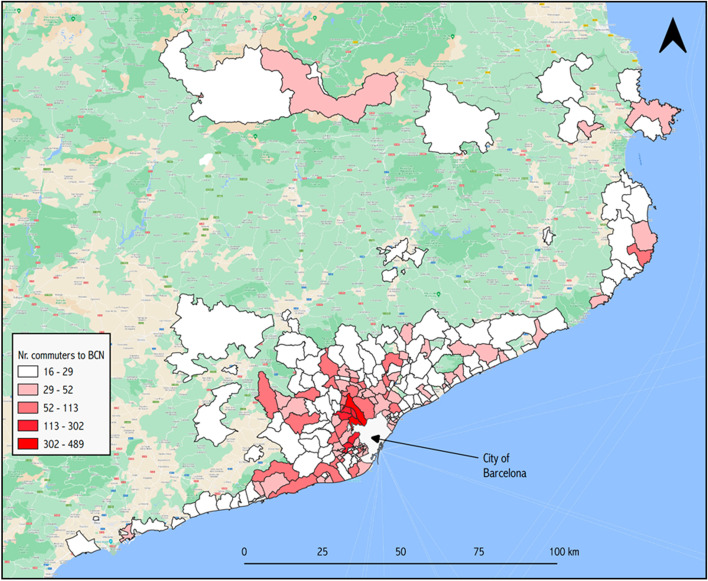
Barcelona’s commuting zone

**Fig. 2:**
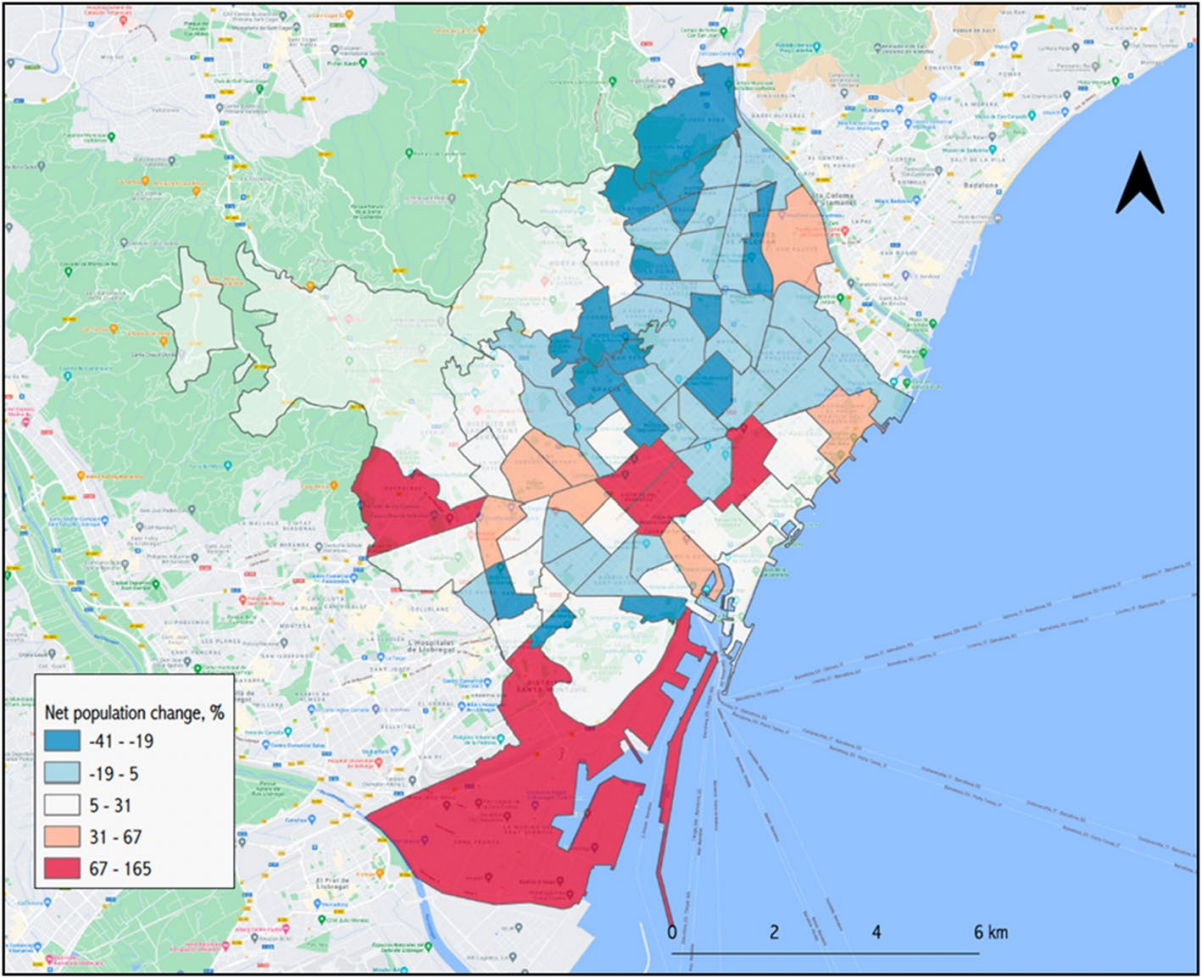
Net population change during work days

**Fig. 3:**
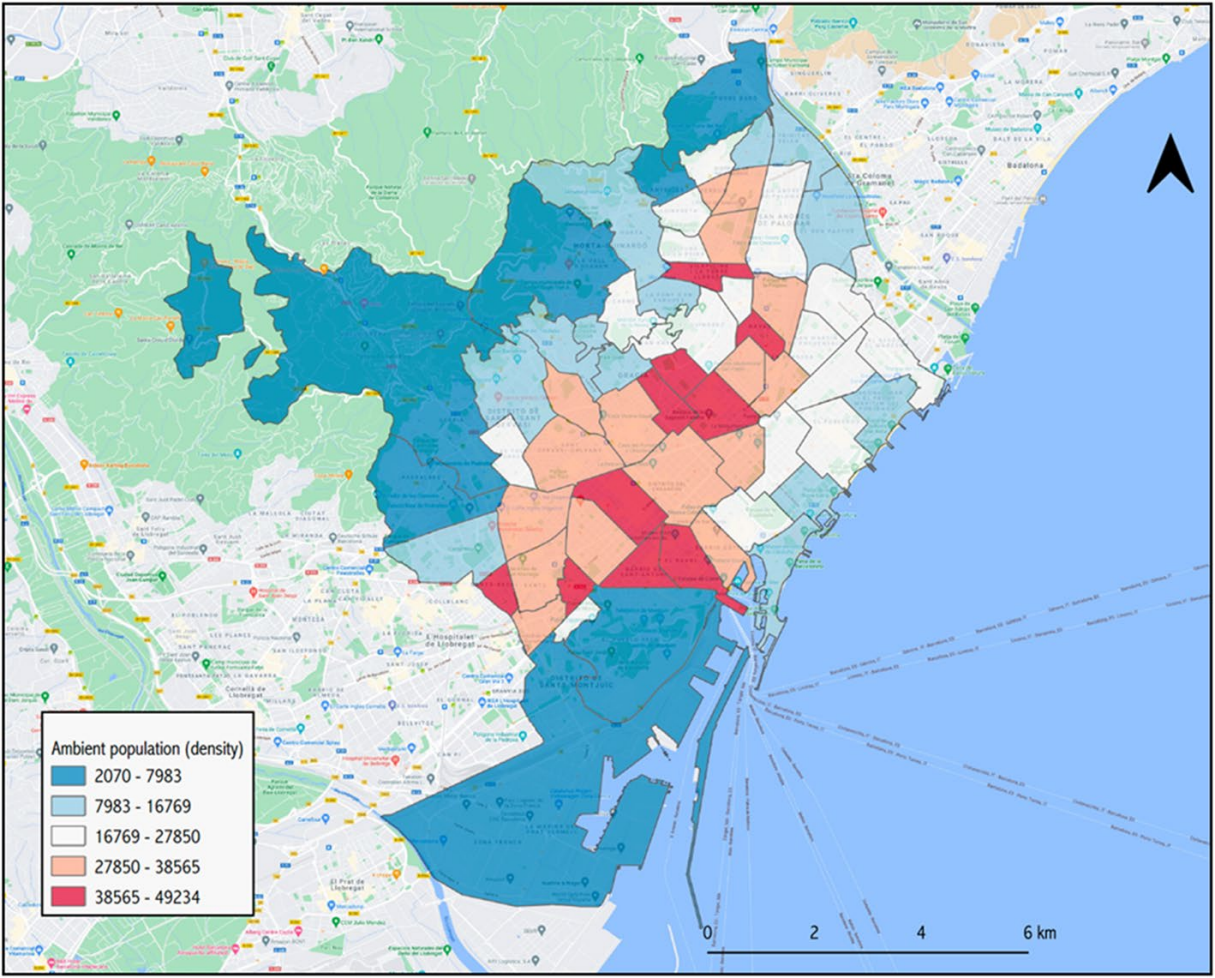
Average density of the ambient population throughout 2019

**Fig. 4:**
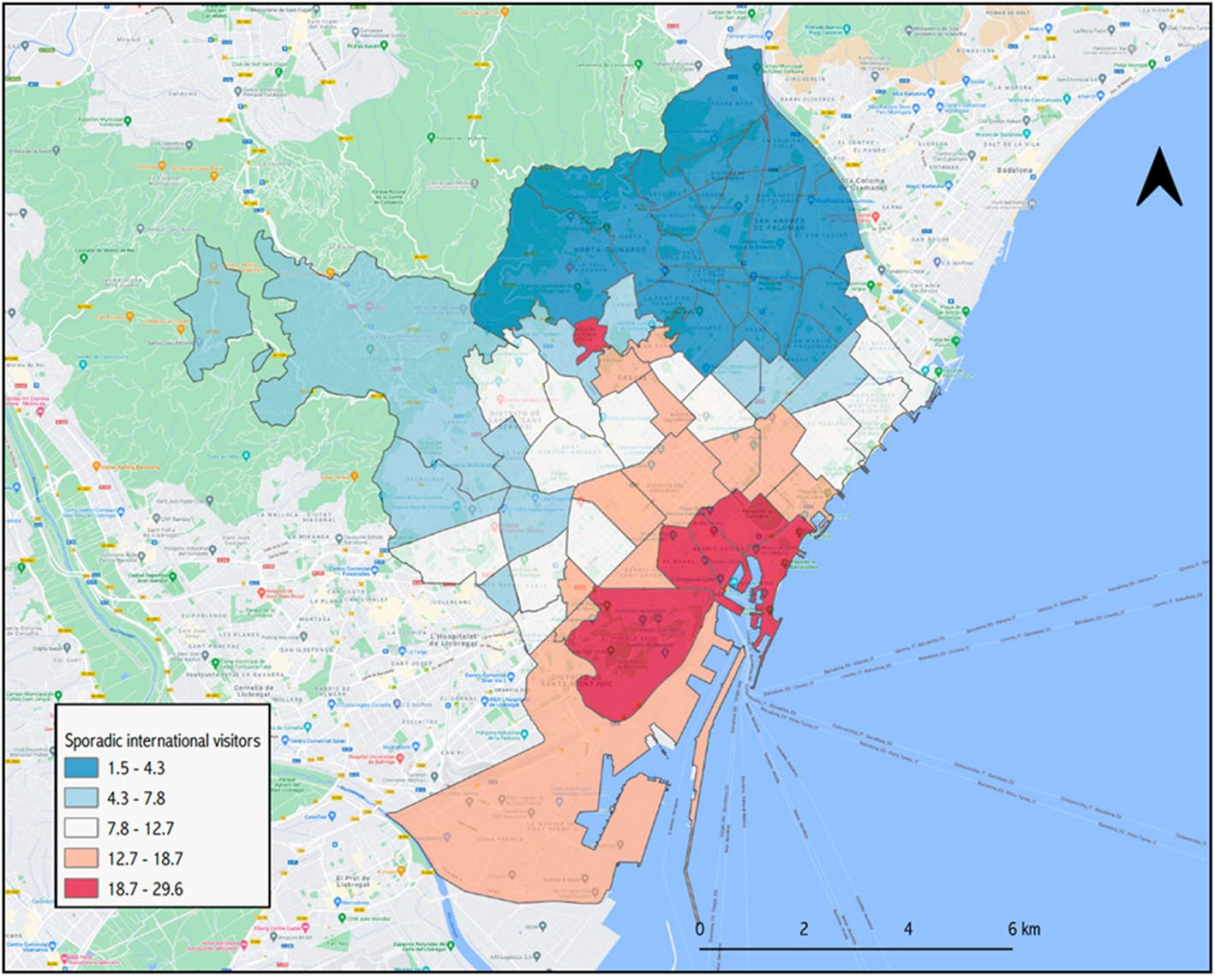
Average percentage of sporadic international users for the same year

On a typical working week, the city of Barcelona receives the equivalent of 48.2% of its total population, about 789,150 people, mostly from its metropolitan area, but also extending to other regions of Catalonia. Figure 1 shows the commuting zone for the reference week of 18–21 November 2019. Looking at the difference between people moving in and people moving out, the city increases its population by 10.4% on workdays. As Fig. 2 shows, however, there are important variations within the city, with some areas seeing their population more than double (coloured in red), while working-class neighbourhoods in the north-east of the city may lose up to 40% of their residents between 10:00 and 18:00 of a working day. As for Figs. 3 and 4, they allow the density of the ambient population to be visualized, averaged throughout 2019, and show the mean percentage of sporadic international visitors for the same reference year. In the first case, higher values correspond to neighbourhoods in the inner-city district of *Ciutat Vella* (including *El Raval*) with an average of 43,498 people per km^2^ occupying public space in 2019 as opposed to a city mean of 25,091, or to areas in the proximity of big transport nodes, for instance, *La Sagrera* or *Estació de Sants*. In the second case, visitors’ patterns closely match tourist attractions in the city. The neighbourhood of *Barceloneta*, famous for its beaches, and *El Gòtic*, situated on the left side of *Las Ramblas*, come at the top of these rankings, with 29.5 and 26.4% of sporadic international visitors in 2019 respectively.

### Model Performance and Output

Although not shown here, our analysis suggest that negative binomial models offer a better fit with the data than Poisson models due to the overdispersal in the counts of robbery. As noted above, we fitted models using logarithmic transformations of the counts (as an alternative to the negative binomial models). These models exhibited significant spatial autocorrelation in the residuals (for the full OLS models, the observed Moran’s *I* were 0.19, 0.11, and 0.14 on weekdays, holidays, and weekends respectively), so we tested the fit of spatial Durbin models, spatial error, and spatial lag models. For two of the outcomes (those with the smallest counts), the Lagrange multipliers were inconclusive (probably a function of the small sample sizes), whereas the AIC and BIC statistics were almost indistinguishable for the simple OLS model and the best performing spatial model for each outcome (see Table 5 in the Appendix). In any case, the pattern of results was substantially the same as we encountered when fitting the negative binomial models (see Table 6 and accompanying notes). Thus, for the rest of the paper, we focus only on describing the results from the negative binomial models.

The results of our negative binomial models presented in Table 2 reveal that values of the dispersion parameter (*θ*) decrease across the models, indicating a reduction in the over-dispersion of the outcome variable. Both the AIC and the BIC, as well as the likelihood ratio test of the nested models, also favour the fully specified models. Looking at the NBM coefficients, a first result refers to the significant and positive effect of our variable of demographic pressure, which is consistent regardless to of daily and seasonal variations in the occurrence of robberies. This finding indicates that in areas where the ambient population outnumber the resident population, the risk of victimization increases for residents. Additionally, if we narrow our focus on non-workdays, the model’s output suggests that a higher density of international visitors in these areas on its own plays a role in the risk of residents’ becoming victims of crime. Conversely, the density of the local ambient population has no effect in the model, and even has a negative sign during weekend, when the proportion of commuters is supposedly lower. We can only speculate about what this means. Perhaps offenders are attracted to these areas because of the increased tourist presence, but once in these areas they also target the non-tourist population.

**Table 2:** NBM outputs with robbery against residents as the DV

Variables	Weekdays (10:00–18:00)			Weekends			Public holidays		
	Model 1a	Model 2a	Model 3a	Model 1b	Model 2b	Model 3b	Model 1c	Model 2c	Model 3c
Exposed population									
Local ambient population (density)	.16 (.10)	.15 (.12)	.07 (.10)	− .15 (.11)	− .16 (.13)	− .10 (.11)	.01 (.11)	− .01 (.12)	.06 (.10)
Sporadic international visitors (density)	.18 (.10)	.13 (.11)	− .06 (.11)	.53*** (.13)	.50*** (.13)	.18 (.13)	.38** (.12)	.34** (.12)	.01 (.11)
Demographic pressure	.26** (.10)	.19 (.10)	.26*** (.07)	.32** (.11)	.18 (.13)	.27** (.09)	.36** (.10)	.23 (.12)	.23* (.09)
Built environment									
Land-use mix		.10 (.10)	.16* (.08)		.13 (.10)	.21* (.10)		.13 (.10)	.14 (.09)
Commercial-to-residential ratio		.22* (.11)	.11 (.07)		.26 (.13)	.10 (.09)		.29* (.12)	.17* (.08)
Control variables									
Residential instability index			.24* (.10)			.29* (.11)			.29** (.10)
Income quintile share ratio			.02 (.08)			.10 (.11)			.08 (.10)
Age (*x̄*)			.10 (.08)			.05 (.10)			− .07 (.10)
Resident population size			.48*** (.07)			.45*** (.09)			.38*** (.08)
Log likelihood	− 263.7	− 261.7	− 238.8	− 308.8	− 306.9	− 288.4	− 231.3	− 228.5	− 210.9
AIC	537.4	537.5	499.6	627.7	627.9	598.8	472.6	471.1	443.9
BIC	548.1	552.5	523.2	638.5	642.9	622.4	483.3	486.1	467.4
Dispersion parameter (θ)	.392	.366	.153	.523	.493	.277	.426	.386	.185

^***^p < .001, **p < .01, and *p < .05

As for variables in the built environment, the most consistent finding refers to the relationship between the ratio of commercial-to-residential land-use and the risk of robbery, although when looking at full models (3a, 3b, and 3c), its effect is statistically significant only during holidays. A greater mixture of land uses is also positively correlated with robbery, possibly because it implies a greater concentration of points of interest and leisure facilities.

As expected, the size of the resident population is a significant predictor of the risk of being the victim of a robbery in Barcelona. The model also depicts a significant and positive effect of the residential stability index on the outcome variable, which is in line with social disorganization theories.

Taking the literature on tourism gentrification into account, we run a follow-up analysis to verify whether there might be a relationship between the density of international tourists and the residential instability, which could in turn influence the distribution of robberies. This assumption was grounded on the idea that a greater presence of tourists is likely to be associated with the spread of vacation rentals to the detriment of long-term residents on the one hand and to socio-environmental concerns (excessive noise, alcohol consumption, overcrowding, etc.) and the desire to change residence on the other. Therefore, we implemented a test for the indirect effects (mediation analysis). The results provide support to the mediation hypothesis by revealing that the index of residential instability significantly mediates the relationship between the density of tourists and the risk of robbery against residents in all models, during weekdays (*β*_ind_ = 0.17; S.E. = 0.08; *p* < 0.05), weekends (*β*_ind_ = 0.17; S.E. = 0.07; *p* < 0.05), and public holidays (*β*_ind_ = 0.15; S.E. = 0.06; *p* < 0.05). In other words, this finding suggests that tourism massification is associated with residential instability in Barcelona, which in turn may lead to a greater risk of residents being involved as the victims of a robbery, mostly likely as the consequence of decreasing levels of social control in over-touristed areas of the city. Exploring this in full would require an analytical approach transcending the limits of a cross-sectional analysis like the one presented here.

Robustness checks do not seem to alter the substantive conclusions from above. A close look at Table 3 in the Appendix reveals that just as for robbery, the risk of theft for residents is greater when we observe increases in the density of sporadic international visitors, the demographic pressure on the resident population, and the residential instability index. Conversely, if the focus is on non-resident victims, the density of sporadic international visitors is nearly the only predictor that matters. Interestingly, the sign of the relationship between the density of the local ambient population and the targeting of tourists is negative, which may indicate that the risk of becoming the victim of a theft or a robbery for a tourist is significantly higher when they are concentrated in tourism-only enclaves with very low densities of residents. The use of an alternative measure of the ambient population keeps the results mostly unchanged. The approach in this case is slightly different, as the dependent variables represent the average sum of robbery and theft for the whole year, and the measure of the ambient population based on the OpenCelliD database does not allow us to distinguish locals from tourists. However, the results in Table 4 largely endorse previous conclusions by stressing the key role of demographic pressure and residential instability on the temporal and spatial patterning of crime in Barcelona.

## Conclusions

According to Battin and Crowl (2017, p. 140), “contemporary neighbourhood development policies, urban sprawl trends, and advancing theoretical understanding prompt a re-examination of the relationship between population density and crime.” This research has supported this re-examination by focusing on the case of Barcelona, Spain. It adds to the previous literature in two ways: first, by exploring the link between urban mobilities and crime, an emerging field that requires international testing, and second, by estimating the effect of spatial concentrations of residents and non-residents on the risk of becoming a victim of robbery. In this regard, this study reinforces existing efforts by providing a more accurate measure of the ambient population, while its originality lies in analysing separately the influence of mobilities undertaken by regulars (i.e., residents and commuters) as opposed to outsiders (i.e., visitors from abroad). However, it also introduces a nuance compared to previous attempts to compute the size of the “exposed population-at-risk” (Haleem et al., 2021) as a combination of transient populations and residents by isolating the impact of a specific transient population sub-group. The rationale behind our approach was two-fold. On the one hand, we assumed that people who use public spaces can perform active role as offenders, victims, or guardians, but also that the uncontrolled influx of international visitors might alter the social composition of an area, thus undermining effective protection and mechanisms of social control. On the other hand, the active performance of these roles is highly dependent on urban rhythms, and we can therefore reasonably expect that the exposed population will increase its size during rush hours when residents are more likely to be out of their homes and the flow of workers and passers-by is more intense.

We found that the presence of international visitors is a consistent explanatory variable of the likelihood of residents becoming victims of robbery, especially outside work-days (i.e., at weekends, in the summer, and on Christmas holidays). The models also depict a connection between the presence of tourists and the residential instability of the host neighbourhoods, which goes in the same direction as previous analyses of the negative externalities of tourism in Barcelona (Cocola-Gant & López-Gay, 2020; Garcia-López et al., 2020; Maldonado-Guzmán, 2020). The sustained and massive influx of visitors is changing the social composition of neighbourhoods in Barcelona. Taking social disorganization theory (Sampson & Groves, 1989; Shaw & McKay, 1942) into account, the decrease in residential stability is de facto an indirect measure of growing social disinvestment, which is reducing social control and opening up opportunities for crime to occur. This seems to be especially the case in areas where land-use is skewed towards commercial facilities, to the detriment of residential uses.

As for the relationship between the density of the ambient population and robbery against residents, interpretation of the NBM coefficients suggests that it has some effect on the dependent variable, but that this is negligeable after controlling for the presence of international tourists and the characteristics of the social landscape. These findings strengthen the interpretation according to which the social context is the key to understanding under what conditions population density may or may not have an effect on crime. That is, it would be easy to assume that, even if a neighbourhood has a relatively uniform level of density, a few areas within it might be overcrowded, especially in the proximity of tourist attractions and points of interest. We might interpret built environment variables in our model along the same lines. A greater mix of land uses, which the literature associates with greater walkability (Frank et al., 2006) and a wider range of services, was found to be positively correlated with robbery, especially during non-work days, possibly because it attracts more visitors.

Our results also reveal an additional layer of complexity, as they allow the influence of two sources of social disorganization to be measured at the neighbourhood level in Barcelona. First, as we mentioned above, residential volatility represents a structural source of social disorganization that significantly increases the risk of becoming a robbery victim. Second, non-stationary densities and demographic pressures on the resident population represent a dynamic source of social disorganization. In fact, in those areas where outsiders outnumber regular users, the number of residents becoming robbery victims is significantly higher. This finding deserves further testing, which falls outside the scope and data availability of the present analysis. However, possible refinements in our modelling approach would open up new avenues for investigation based on a more detailed breakdown into different categories of transient populations (national versus international tourists, commuters, shoppers, residents from adjacent neighbourhoods, and so forth), or using non-linear models to test the robbery-density hypothesis (Angel, 1968; Clarke et al., 1996; Newton, 2018) by looking at different transient population densities. Likewise, it seems crucial to devise alternative measures that could enable a closer examination of divergent patterns of occupancy and social interaction in the public and private spaces to which people have access (commercial buildings, recreational facilities, parking lots, etc.).

As with any study of a single city, the results should not be generalized to other contexts, even though they might inform research on urban areas that are affected by similar issues of overcrowding and excessive tourism. This study is also limited by its reduced time frame, the relatively small sample size, and the cross-sectional nature of the analysis. These limitations result for the most part from the nature of the available data. For the same reason, it was not possible to study the relationship between mobility flows and robbery patterns outside the 10:00–18:00 h period, which should also be regarded as a possible source of bias overlooking intra-daily and seasonal variations in the use of public spaces in Barcelona. Future research is needed to fill these gaps, as well as to fine-tune current measures of human mobility at finer geographical and temporal resolutions. Despite these limitations, the results are consistently aligned with those of other scholars who have suggested that outsider visitors undermine the social integration of the host neighbourhoods, thus reducing the willingness of local residents to cast their “eyes on the street” (Jacob, 1961). Our findings have strong implications for current post-COVID-19 urban planning and crime prevention debates in at least two regards. First, they warn about crime as an externality of tourism and the pitfalls of going back to business as usual. Pre-pandemic tourism, and more specifically short-term rentals, may put neighbourhoods under pressure, as they reduce the availability of affordable housing and push residents to move out. Therefore, measures to avert a housing crisis and retain residents in tourism destinations should perhaps rank as top priorities. Second, the model’s outputs indicate that in itself density is not a sufficient explanation for why and where crime occurs in Barcelona. Rather, they suggest that, first, the presence of tourists contributes to sharpening a structural problem of overcrowding, which comes with negative consequences in terms of a greater exposure of residents to the risk of robbery. Second, traditional density measures tell us very little about the social composition of the population that is using or crossing urban space, which might be one of the reasons why the exact nature of the relationship between population densities and crime has proved controversial in the literature. Our results point to the density of social ties, and not just to the proximity of people, as a precondition for the actual provision of informal social control. Given that the social restrictions and health precautions imposed by the pandemic have engendered new rules of interaction, questioning traditional meanings, and uses of public space, the reactivation of tourism with its corollary of place massification could increase the social distance between residents and tourists further. Close monitoring of non-stationary density and its social costs, improving the regulation of spatial occupancy, redistributing the economic benefits of tourism to society at large, and involving local communities in local decision-making are among possible solutions to excessive tourism in post-pandemic scenarios.

## Data Availability

The data that support the findings of this study are not publicly available due to third party restrictions.

## Appendix 1. Robustness checks.

Tables 3, 4, 5 and 6

**Table 3:** Robustness checks (sets 1 and 2)

Variables	DV = theft against residents			DV = robbery against tourists			DV = theft against tourists		
	Weekdays	Weekends	Public holidays	Weekdays	Weekends	Public holidays	Weekdays	Weekends	Public holidays
Local ambient population (density)	.03 (.10)	− .14 (.13)	− .07 (.13)	** − 2.14*** (.52)**	** − 1.87*** (.43)**	** − 1.68*** (.48)**	** − 1.52*** (.27)**	** − 1.03*** (.23)**	** − 1.25*** (.32)**
Sporadic international visitors (density)	.23 (.12)	**.33* (.16)**	**.39* (.15)**	**2.37*** (.58)**	**2.20*** (.45)**	**2.04*** (.51)**	**2.56*** (.34)**	**1.65*** (.30)**	**2.11*** (.42)**
Demographic pressure	**.30*** (.08)**	**.34** (.11)**	**.37** (.12)**	.08 (.26)	**.72** (.27)**	.42 (.28)	.04 (.21)	.28 (.20)	.23 (.27)
Land-use mix	.16 (.08)	.23 (.12)	.16 (.13)	.46 (.35)	**.83** (.32)**	**.66* (.33)**	.01 (.25)	.32 (.23)	.57 (.31)
Commercial-to-residential ratio	.10 (.07)	.14 (.12)	.02 (.11)	.13 (.30)	− .28 (.29)	.09 (.26)	− .14 (.19)	− .06 (.21)	− .18 (.26)
Residential instability index	**.26** (.09)**	**.41*** (.12)**	**.34** (.12)**	− .62 (.46)	− .12 (.24)	− .09 (.27)	− .29 (.23)	.34 (.18)	.17 (.24)
Income quintile share ratio	.06 (.09)	− .01 (.15)	− .05 (.15)	.47 (.38)	.36 (.29)	.10 (.33)	.06 (.30)	.08 (.29)	− .13 (.41)
Age (*x̄*)	.04 (.08)	− .02 (.12)	− .13 (.12)	.08 (.32)	.11 (.27)	− .23 (.24)	− .01 (.20)	.07 (.20)	− .25 (.25)
Resident population size	**.47*** (.07)**	**.42*** (.11)**	**.41*** (.11)**	**1.41*** (.39)**	**1.20*** (.30)**	**.77* (.33)**	**.83*** (.23)**	**.53** (.19)**	**.83** (.30)**
Log likelihood	− 313.8	− 329.7	− 273.3	− 81.6	− 101.0	− 89.1	− 217.5	− 234.8	− 191.5
AIC	649.6	681.4	568.7	185.3	224.0	200.2	457.1	491.7	405.0
BIC	673.2	705.0	592.3	208.9	247.6	223.8	480.7	515.3	428.5
Dispersion parameter (θ)	.195	.443	.414	1.128	.778	.992	1.197	1.005	1.594

^***^*p* < .001, ***p* < .01, and **p* < .05

**Table 4:** Robustness checks (set 3). NBM outputs with a different proxy measure of the ambient population (OpenCelliD)

Variables	DV = robbery against residents	DV = theft against residents	DV = robbery against tourists	DV = theft against tourists
Exposed population				
OpenCelliD (density)	.14 (.08)	**.32*** (.09)**	**.72** (.27)**	**.86*** (.24)**
Demographic pressure	**.26*** (.07)**	**.36*** (.08)**	**1.22*** (.32)**	**.80*** (.24)**
Built environment				
Land-use mix	**.18* (.07)**	.14 (.08)	.40 (.32)	.29 (.25)
Commercial-to-residential ratio	**.17* (.06)**	.11 (.07)	− .30 (.27)	− .02 (.18)
Control variables				
Residential instability index	**.31*** (.07)**	**.51*** (.08)**	**.92*** (.25)**	**.97*** (.21)**
Income quintile share ratio	− .01 (.09)	− .11 (.10)	− .11 (.38)	.09 (.34)
Age (*x̄*)	.06 (.08)	.01 (.08)	.05 (.29)	.23 (.22)
Resident population size	**.40*** (.07)**	**.37*** (.07)**	.59 (.31)	.10 (.18)
Log likelihood	− 246.1	− 299.1	− 89.4	− 223.2
AIC	512.2	618.1	198.9	466.4
BIC	533.7	639.6	220.3	487.8
Dispersion parameter (θ)	.152	.206	1.003	1.225

^***^*p* < .001, ***p* < .01, and **p* < .05

**Table 5:** Assessment of best spatial model

	Weekdays	Holidays	Weekend
Lagrange multiplier Error (*p* value)	0.02	0.14	0.07
Lagrange multiplier lag (*p* value)	0.02	0–09	0.02
Robust LM error (*p* value)	0.27	0.65	0.65
Robust LM lag (*p* value)	0.30	0.33	0.15
AIC OLS model	129.17	87.89	85.30
BIC OLS model	152.75	111.46	108.88
AIC best spatial model^1^	125.62	87.54	83.40
BIC best spatial model^1^	151.34	113.25	109.12

^1^Meaning the one with the lowest AIC and BIC

**Table 6:** Spatial error models of logarithmic transformation of count of robbery against residents

Variables	Weekdays	Weekend	Public holidays
Exposed population			
Local ambient population (density)	.07 (.09)	− .06 (.11)	.01 (.08)
Sporadic international visitors (density)	− .08 (.10)	.15 (.12)	.05 (.09)
Demographic pressure	**.24*** (.06)**	**.25** (.08)**	**.17* (.07)**
Built environment			
Land-use mix	.12 (.07)	**.29** (.10)**	.11 (.08)
Commercial-to-residential ratio	**.13* (.06)**	**.19* (.09)**	**.19** (.07)**
Control variables			
Residential instability index	**.18* (.09)**	**.23* (.11)**	**.21* (.09)**
Income quintile share ratio	− **.**00 (.08)	.18 (.11)	**.**06 (.08)
Age (*x̄*)	.09 (.08)	.14 (.10)	− .03 (.08)
Resident population size	**.44*** (.06)**	**.48*** (.08)**	**.32*** (.06)**

Note 1. The spatial Durbin model was not appropriate in any case, and although for two dependent variables, the diagnostic statistics were not helpful to select between the non-spatial, the error or the lag model, for parsimony and to show the impact of taking into account spatial autocorrelation, we show here the spatial error models (in which the coefficients are also easier to interpret). Note 2. As in the NB models, demographic pressure, residential instability, and residential population are significant across the three models. For public holidays, the same variables emerge as significant in the spatial error and the NB model. For weekdays, it is the commercial to residential ratio and not land use mix that emerges as significant. And for weekends, unlike in the NB model, commercial to residential ratio is significant in addition to land-use mix

^***^*p* < .001), ***p* < .01, and **p* < .05
